# Neurocognitive impairment and health-related quality of life among people living with Human Immunodeficiency Virus (HIV)

**DOI:** 10.1371/journal.pone.0248802

**Published:** 2021-04-01

**Authors:** Philip S. Amara, Zaeema Naveed, Christopher S. Wichman, Howard S. Fox, Lorena Baccaglini

**Affiliations:** 1 Department of Epidemiology, University of Nebraska Medical Center, Omaha, Nebraska, United States of America; 2 Department of Biostatistics, University of Nebraska Medical Center, Omaha, Nebraska, United States of America; 3 Department of Neurological Sciences, University of Nebraska Medical Center, Omaha, Nebraska, United States of America; University of New South Wales, AUSTRALIA

## Abstract

The association between HIV-associated neurocognitive impairment (NCI) and health-related quality of life (HRQoL) is not well known. We investigated this association among the CNS (Central Nervous System) HIV Antiretroviral Therapy Effects Research (CHARTER) study participants. We performed factor analysis to distinguish physical and mental HRQoL, followed by general linear models. We analyzed 1,340 HIV participants, including 35.6% with NCI, 77.2% males, 70.5% unemployed, and 42.2% with depression. Impaired participants had lower (worse) mental and physical HRQoL mean scores compared to unimpaired participants. NCI was negatively associated with mental HRQoL in crude (mean difference: -4.38; 95% CI: -6.70 to -2.06) and adjusted analysis (-2.56, -4.83 to -0.30). NCI was also negatively associated with physical HRQoL in unadjusted analysis (-4.62, -7.45 to -1.78), though the association weakened in the adjusted analysis (-2.20, -4.81 to 0.40). The association between NCI and HRQoL was confounded mainly by employment and was partially mediated by depression. These findings suggest that future strategies aimed at improving HRQoL among HIV-infected patients with NCI might benefit from concurrent management of depression.

## Introduction

The introduction of combination antiretroviral therapy (cART) in the late 1990s has resulted in increased longevity of persons living with the Human Immunodeficiency Virus (HIV), changing the status of HIV from a fatal disease to a manageable chronic condition [1]. People living with HIV (PLWH) are at risk for neurocognitive impairment (NCI), with possible negative consequences for their quality of life. In the cART era, HIV-related dementia has declined, but mild to moderate forms of HIV-associated neurocognitive disorders (HAND) remain a problem [2]. With 38 million PLWH worldwide in 2019 [3], health-related quality of life (HRQoL) has become an important health outcome for PLWH. HRQoL is a subjective and multidimensional measure of well-being involving the individual’s physical, mental, emotional, and social functioning [1, 4].

HIV infection and its severity are associated with lower HRQoL and NCI [5–8]. For example, Engelhard et al. reported that PLWH have poorer mental HRQoL than individuals with other comorbidities (i.e., diabetes, or rheumatoid arthritis) [7]. Moore et al. found that successful psychosocial aging is possible in older PLWH, but the HIV+ group in the study had lower physical and mental health functioning and greater psychosocial stress compared to the HIV- group [6]. Moore et al. also found that fewer HIV+ persons met the criteria for successful cognitive aging, defined as having no cognitive or everyday impairment or major depressive disorder, compared to HIV- individuals [8]. However, HIV+ individuals who met those criteria had comparable HRQoL as HIV-individuals.

Other studies have suggested that NCI reduces HRQoL in HIV infection [9–13]. A large Canadian study of HIV+ men found that cognitive performance indirectly affects HRQoL through its impact on cognitive difficulties and the ability to engage in meaningful activities such as recreation, leisure, household management or sports [11]. A longitudinal study conducted in a large sample of HIV+ men found a negative long-term association between cognitive function and HRQoL and showed that changes in cognitive functioning occur before changes in HRQOL [12]. An analysis of 59 PLWH showed a correlation between poorer mental QoL and lower performance on neurocognitive tests for speed of mental processing and flexibility [13]. However, these studies were based on non-US based, male-only or small samples, used self-reported measures of NCI, did not always adjust for confounders, or used different research case definitions or measures [6–13].

Thus, the main objective of this study was to systematically investigate the association between NCI and HRQoL in a large and diverse sample of males and females PLWH in the US.

NCI was measured by global deficit scores (GDS) obtained through a battery of neuropsychological (NP) tests. HRQoL was measured using participants’ responses to the 35-item Medical Outcome HIV Health Survey (MOS-HIV) questionnaire. The questionnaire items can be grouped into eleven scales, and the scales can be further summarized into physical and mental health dimensions. However, prior studies disagree on how to produce summary scores using the eleven scales [14–16]. Thus, a secondary objective of this analysis was to verify that the MOS-HIV questionnaire can be summarized into physical and mental health summary scores for the study population and to determine how the scales should be grouped together using factor analysis. Previous studies have reported that depression is a mediator of the relationship between NCI and HRQoL [10]. We also conducted a simple mediation analysis to test this assumption.

## Materials and methods

### Data source and participants

We analyzed data from the CHARTER study, a prospective, observational study conducted from 2003 to 2015 in three phases [17, 18]. The primary aim of the CHARTER study was “to determine how central and peripheral nervous system complications of HIV are affected by different histories and regimens of antiretroviral therapy” [17, 18]. The CHARTER study was conducted at six University research clinics in the United States: University of California, San Diego, CA (UCSD); Johns Hopkins University, Baltimore, MD; the Mount Sinai School of Medicine, New York, NY, University of Texas Medical Branch, Galveston, TX; University of Washington, Seattle, WA and Washington University, St Louis, MO [2, 17]. Specifically, we analyzed cross-sectional baseline data, which were collected from 2003 to 2007. We selected the baseline study because it provided the data and variables needed to address our research question.

A total of 2,016 HIV patients attending a clinic at a participating university center were screened at baseline and invited to participate in the study, with minimal exclusion criteria (i.e., they declined to participate or could not complete the assessments) [2, 17]. At baseline, all participants were HIV+ and were included irrespective of ARV status, viral load in CSF, duration of HIV infection or neurocognitive complications [18].

**Fig 1:**
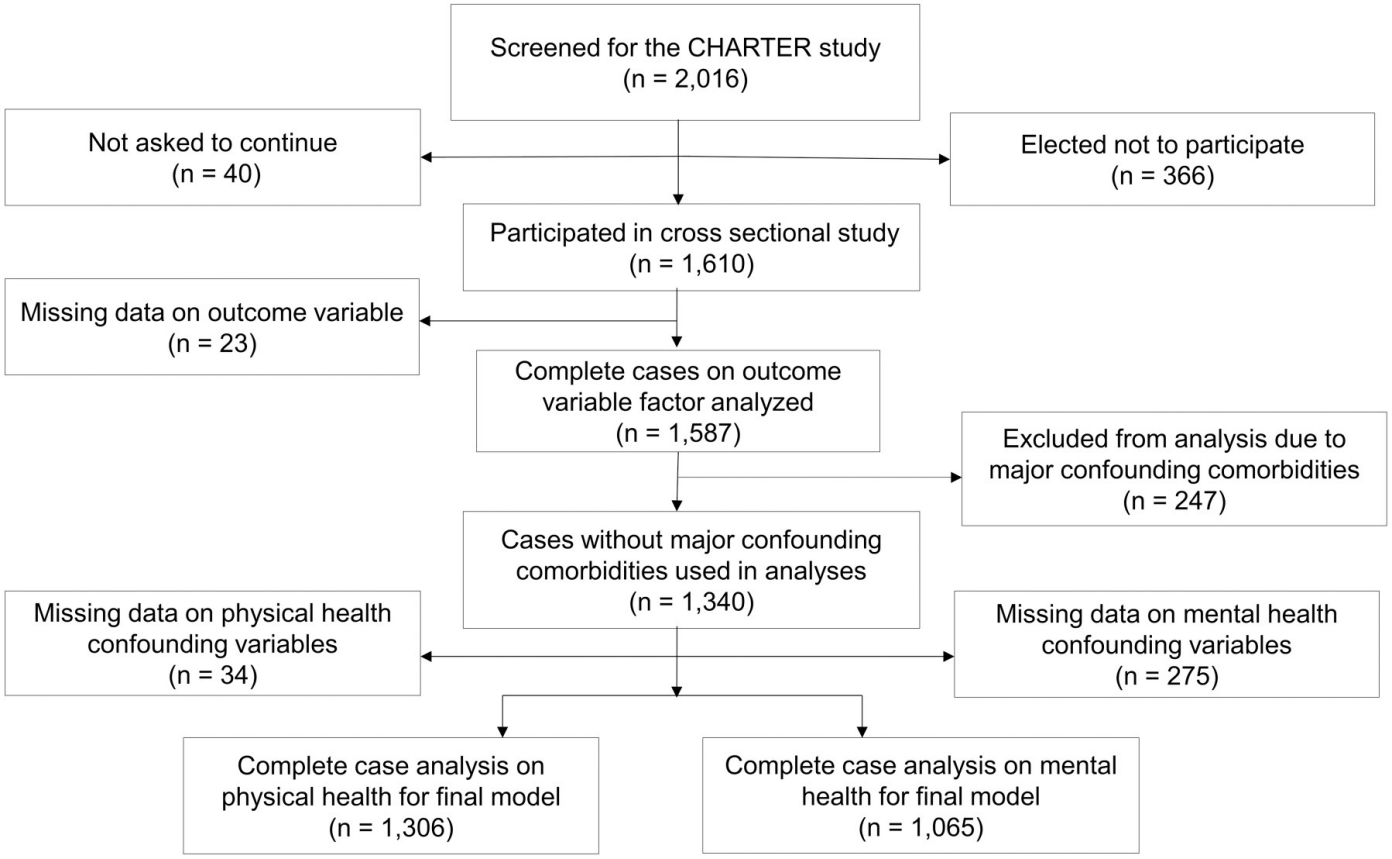
Flow chart showing final samples sizes of CHARTER participants used in study.

Of the participants screened, 40 (2%) were not asked to continue, whereas 366 (18%) declined to participate. Cross-sectional baseline data were collected from 1,610 HIV infected participants. Of these, 1,587 participants had complete data on HRQoL and were included in the factor analysis (Fig 1).

In addition to HIV, several comorbidities can contribute to NCI. CHARTER study’s investigators administered comprehensive NP tests, collected data on comorbid conditions and detailed information on functional limitations. Comorbid conditions included brain trauma, special education problems, reading difficulties, current or lifetime major depression, epilepsy and history of other seizure disorders, substance overdose with complications and psychotic disorders [2]. All participants were classified as having incidental, contributing, or confounding comorbidities. A senior neuropsychologist used standard guidelines in addition to historical and testing data to rate the comorbidity status of participants. A reliability check was conducted by two independent neurologists [2]. Confounding conditions were defined as “conditions that could fully explain significant NCI and currently observed problems with everyday functioning and precluded the attribution of NCI solely to the effects of HIV on the brain” [2]. We excluded participants with major confounding comorbidities or missing data on analytic variables (Fig 1).

### Measurements

The main outcome variables were physical and mental HRQoL. HRQoL was measured by the standardized 35-item MOS-HIV, which is based on patients’ self-reports of their subjective well-being. At the first clinic visit, participants were asked to complete the MOS-HIV using the past four weeks as a reference period. The survey was administered by an interviewer if the participants had trouble reading. The completed questionnaire was reviewed for completeness before the clinical examination. The MOS-HIV contained eleven scales: pain, physical functioning, social functioning, mental health, energy or fatigue, health distress, cognitive functioning, general health, role functioning, quality of life and health transition. Using factor analysis, the MOS-HIV was reduced into physical and mental HRQoL summary scores, which served as the main outcome variables [14].

The main independent variable was HIV-associated NCI, as measured by global deficit scores (GDS). Data to compute NCI were collected using a comprehensive set of NP tests covering seven cognitive domains [2, 19 (S1 Table)]. The tests measured participants’ performance on the speed of information processing, learning, memory, executive function, verbal fluency and working memory, and motor functioning. Multiple tests were administered to measure each domain [2]. Raw test scores for each participant were adjusted for demographic characteristics and converted into T-scores [19]. The T-scores were recoded into deficit scores as follows: T-scores greater than 39 were coded as 0 = normal, scores = 35–39 were coded as 1 = mild impairment, scores = 30–34 were coded as 2 = mild to moderate impairment, scores = 25–29 were coded as 3 = moderate impairment, while scores = 20–24 were coded 4 = moderate to severe impairment and scores less than or equal to 19 were coded as 5 = severe impairment. GDS were calculated by averaging individual deficit scores (0–5) across all NP tests, and participants were classified as neurocognitively impaired if their GDS was greater than or equal to 0.5 [19].

Depression was measured by the Beck Depression Inventory (BDI-II). A score greater than 13 points on the BDI-II was classified as mild to severe depression.

### Statistical analysis

We performed factor analysis to reduce the 11 scales of the MOS-HIV questionnaire into two dimensions, followed by univariable analyses and regression modelling. Data reduction to compute summary scores for the MOS-HIV questionnaire followed a three-step process. First, 11 of the 35 MOS-HIV questionnaire items were reverse coded to ensure that higher scores reflected a more favorable health status. The 35 items were then summarized into 11 scales by adding questionnaire items designed to measure common constructs. The scales were: pain (2 items), physical functioning (6 items), role functioning (2 items), social functioning (1 item), mental health (5 items), energy or fatigue (4 items), health distress (4 items), cognitive functioning (4 items), general health perception (5 items), quality of life (1 item) and health transition (1 item). The scales were transformed into 0–100 scores, with 100 representing the best health status [14]. In the second step, we performed an exploratory factor analysis on a subsample of 794 participants representing half of the full dataset randomly selected from the total sample using the SAS PROC SURVEYSELECT procedure. The exploratory factor analysis procedure used squared multiple correlations as prior communality estimates. We tested a 1, 2, 3 and 4 factor solution. The rotated factor pattern was used to determine what scales loaded on which factor. A scale loaded on physical health and not on mental health if a factor loading of the scale was 0.4 or higher on the physical health factor but less than 0.4 on the mental health factor. Two factors were extracted by the principal factor method followed by a promax rotation. The health transition scale did not load on any factor, so it was not included in the confirmatory factor analysis [20]. In the third step, the extracted factors were validated using confirmatory factor analysis applied to the full dataset of 1,587 participants. A measurement model that describes the relationship between the 2 latent factors and the 10 scales was developed and confirmatory factor analysis was conducted to demonstrate that the model fit [20, 21]. The maximum likelihood method (MLM) was used for parameter estimation.

Regression model development also followed a structured approach. We conducted a comprehensive literature review that informed the development of a causal directed acyclic graph (DAG) in DAGitty (dagitty.net; version 3.0), exhibiting the theoretical relationships among potential confounders of the relationship between NCI and HRQoL. Univariable analyses using independent sample t-tests, Mann-Whitney U tests, Pearson correlation coefficients, and one-way analysis of variance (ANOVA) were conducted to investigate the crude relationship between NCI or HRQoL and candidate confounders. We tested for normality using the univariate procedure in SAS version 9.4. Variables associated with both NCI and HRQoL at 2-sided α = 0.10 were considered potential confounders. The final confounding assessment was conducted in two steps [22]. We first estimated the mean difference of the relationship between HRQoL (physical and mental) and NCI for a reduced model that included NCI, age, sex at birth and race/ethnicity. We then recorded the change in the estimate for the association between NCI and HRQoL when potential confounders were added to the model. If the percent change in the estimate for the reduced model compared to the model with the added variable was greater than 10%, we considered the variable a confounder and included it in the model.

Depression was not included in the models because we assumed, based on evidence from previous studies, that depression was a mediator of the relationship between NCI and HRQoL [10]. To test this assumption, we used a single mediation analysis [10]. To establish mediation, we regressed depression on NCI, HRQoL on NCI, HRQoL on NCI controlling for depression, and depression on HRQoL [10, 23]. SAS version 9.4 (SAS Institute Inc., Cary, NC) was used for all the analyses.

All CHARTER study procedures involving human participants were in accordance with the ethical standards of the institutional and/or national research committee and with the 1964 Helsinki declaration and its later amendments or comparable ethical standards. The Institutional Review Boards (IRB) at the six participating Universities, i.e., the University of California, San Diego, CA (UCSD); Johns Hopkins University, Baltimore, MD; the Mount Sinai School of Medicine, New York, NY, University of Texas Medical Branch, Galveston, TX; University of Washington, Seattle, WA and Washington University, St Louis, MO reviewed and approved the study; the analyses conducted by the Data Coordinating Center were approved by the University of Nebraska Medical Center’s IRB (Protocol #282-13-EP).

## Results

### Participants’ characteristics

A total of 1,587 participants had complete data on the outcome variable. After excluding 247 (16%) participants with severe comorbidities, the remaining 1,340 participants had incidental comorbidities (54%) or contributing comorbidities (30%). Comorbid conditions included brain trauma, epilepsy, low reading levels, major depression, lifetime or current substance use or alcohol disorder. The final sample included 77% males, 47% non-Hispanic Black or African Americans, 41.1% non-Hispanic Whites and 71% unemployed participants with a mean age of 43.0 years (standard deviation, SD = 8.65). Approximately 42% of the participants were neurocognitively impaired, as determined by their GDS scores.

### Factor structure of the MOS-HIV health survey

The MOS-HIV could be summarized into physical and mental health components. A single factor solution accounted for 90% of the variance and the rotated factor pattern demonstrated a simple structure. The variables that loaded on the single structure had high factor loadings (above the cutoff value of 40%), suggesting that a single factor was admissible. However, a two-factor solution accounted for the full variance, with the second factor accounting for 10% of the total variance. A three-factor solution did not show a simple structure, and only two factors were extracted and interpretable based on a four-factor solution [20]. Thus, two factors were extracted by the principal factor method, followed by a promax rotation [20].

Five scales (cognitive function, health distress, energy/fatigue, quality of life and mental health) loaded on mental health (S3 Table) and five scales (pain perception, physical function, role function, general health and social function) loaded on physical health (S4 Table). The health transition scale did not load on any factor, so it was not included in the confirmatory factor analysis. The eigenvalues, the proportion of variance explained, and the scree plots combined with the rotated factor pattern showed that the MOS-HIV questionnaire could be summarized into two factors: mental health and physical health. Pearson correlation coefficients (r) for the 10 scales of the MOS-HIV questionnaire ranged from r = 0.33 to 0.66, indicating a relatively strong positive association among the variables (Table 1).

**Table 1:** Means, standard deviations and Pearson correlation coefficients for the health-related quality of life (HRQoL) scales of the MOS-HIV health survey (n = 1,587)^a^.

***No***	***HRQoL scales***^b^	***Mean***^c^	***SD***^d^	***HRQoL scales***									
				1	2	3	4	5	6	7	8	9	10
*1*	Mental health	66.55	21.66	1									
*2*	Energy/Fatigue	53.28	22.21	0.66	1								
*3*	Health distress	72.10	26.33	0.66	0.57	1							
*4*	Cognitive function	72.13	23.80	0.59	0.56	0.59	1						
*5*	Quality of life	64.44	22.06	0.59	0.55	0.50	0.43	1					
*6*	Pain	66.24	26.73	0.42	0.52	0.50	0.41	0.42	1				
*7*	Physical function	69.02	27.47	0.35	0.49	0.45	0.43	0.33	0.58	1			
*8*	Role function	54.09	45.32	0.38	0.49	0.47	0.41	0.36	0.51	0.55	1		
*9*	Social function	74.12	28.45	0.53	0.56	0.60	0.54	0.44	0.55	0.58	0.56	1	
*10*	General health	48.25	26.91	0.52	0.59	0.60	0.46	0.56	0.58	0.52	0.56	0.54	1

^a^MOS, Medical Outcome Survey Questionnaire;

^b^The scale health transition did not load on any factor;

^c^Higher mean scores indicate better quality of life perception, lower pain and limitations in role function;

^d^SD means Standard Deviation.

**Fig 2:**
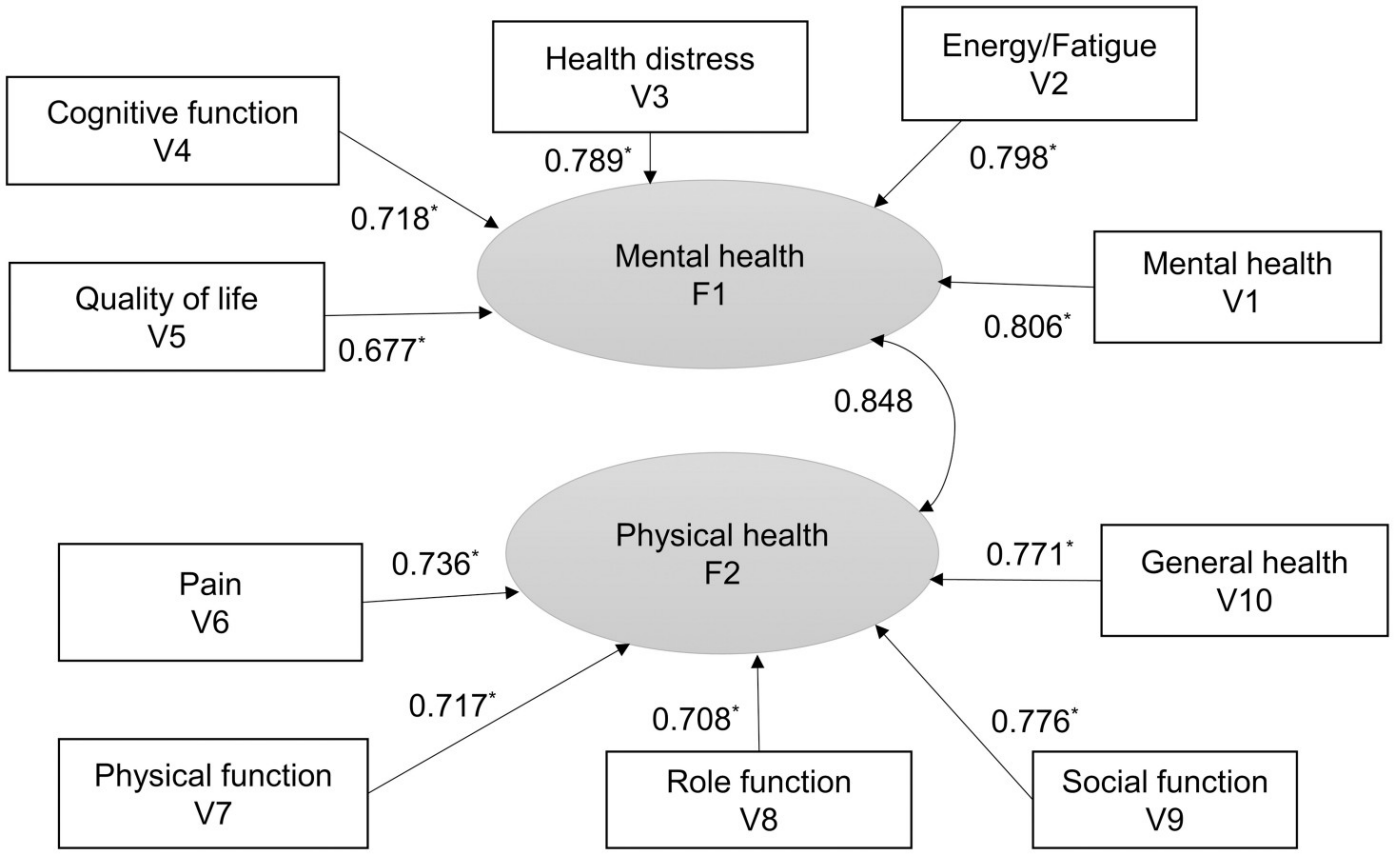
Path model of health-related quality of life of CHARTER participants (n = 1,587). Single-headed arrows show standardized path coefficients; Curved double-headed arrow shows covariance for two factors. * p < 0.001. F1, Factor 1; F2, Factor 2; V1…V10, represent variable names.

All the standardized path coefficients were greater than 0.65, providing support for the convergent validity of the scales (Fig 2 [20]).

### Differences in HRQoL between HIV-positive neurocognitively impaired and unimpaired participants

Our analysis based on the DAG revealed that age, employment, CD4 nadir, psychotropic drug use, substance abuse, central nervous system integrity (represented by cerebrospinal fluid (CSF) glucose, CSF total protein, HIV viral load in plasma and CSF) were potential confounders of the relationship between NCI and HRQoL (S1 Fig) while depression was identified as a mediator [22].

The results of t-tests showed that unimpaired participants had higher (i.e., better) physical (p = 0.001) and mental HRQoL composite scores (p < 0.001) than impaired participants (Tables 2 and 3). Specifically, unimpaired participants reported higher mean scores for mental health (p = 0.005), energy/fatigue (p = 0.003), health distress (p = 0.003), cognitive function (p < 0.001), physical function (p < 0.001), role function (p = 0.004), and social function (p = 0.007) compared to impaired participants. However, impaired, and unimpaired participants had similar quality of life perception (p = 0.156), pain (p = 0.181) and general health perception (p = 0.160).

**Table 2:** Relationship between neurocognitive impairment and mental health-related quality of life (HRQoL) of CHARTER participants (n = 1,065).

Variable	n (%)^a^	Mental HRQoL^b^ composite scores Mean (SD)^c^	Unadjusted mean difference (95% CI)^d^	Adjusted mean difference (95% CI)^e^
**Neurocognitive impairment (GDS)**^f^			p < 0.002	
No	679 (63.8)	65.82 (18.82)	Ref	ref
Yes	386 (36.2)	61.45 (18.11)	-4.38 (-6.70 to -2.06)	-2.56 (-4.83 to -0.30)
**Gender at birth**			p = 0.273	
Male	807 (75.8)	64.59 (18.35)	Ref	ref
Female	258 (24.2)	63.12 (19.66)	-1.47 (-4.09 to 1.15)	-2.14 (-4.75 to 0.46)
**Age (years)**			p = 0.571	
≤ 39	321 (30.2)	64.87 (19.08)	Ref	ref
40–49	504 (47.3)	64.05 (18.38)	-0.82 (-3.44 to 1.80)	-0.11 (-2.65 to 2.42)
50–59	212 (19.9)	63.24 (18.35)	-1.63 (-4.88 to 1.61)	-0.09 (-3.22 to 3.04)
≥ 60	28 (2.6)	67.74 (21.84)	2.86 (-4.36 to 10.09)	4.23 (-2.68 to 11.14)
**Race/Ethnicity**			p = 0.001	
Black/African American	479 (45.0)	66.75 (18.07)	Ref	ref
White	462 (43.4)	62.05 (18.97)	-4.69 (-7.07 to -2.32)	-5.86 (-8.28 to -3.44)
Hispanic	99 (9.3)	62.58 (17.56)	-4.17 (-8.19 to -0.15)	-4.17 (-8.09 to -0.25)
Other	25 (2.3)	63.09 (23.76)	-3.66 (-11.13 to 3.81)	-5.05 (-12.25 to 2.15)
**Employment**			p < 0.001	
Full time	152 (14.2)	71.96 (16.53)	Ref	ref
Part-time	137 (12.9)	65.50 (18.57)	-6.46 (-10.71 to -2.20)	-6.64 (-10.78 to -2.50)
Unemployed	776 (72.9)	62.50 (18.71)	-9.46 (-12.66 to -6.26)	-8.96 (-12.17 to -5.75)
**Current psychotropic medication use**			p < 0.001	
No	310 (29.1)	71.28 (16.21)	Ref	ref
Yes	755 (70.9)	61.34 (18.86)	-9.93 (-12.33 to -7.53)	-8.21 (-10.63 to -5.80)

^a^n, sample size with percentage of respondents in bracket; ref, means the reference category;

^b^HRQoL, Health-related quality of life; Scores vary from 0–100 with higher scores indicating better quality of life;

^c^SD, Standard deviation;

^d^CI, Confidence interval;

^e^Adjusted for other covariates in the table using general linear model analysis;

^f^GDS, global deficit scores, used to determine impairment.

**Table 3:** Relationship between HIV-associated neurocognitive impairment and physical health-related quality of life (HRQoL) of CHARTER participants (n = 1,306).

Variable	n (%)^a^	Physical HRQoL^b^ composite scores Mean (SD)^c^	Unadjusted mean difference (95% CI)^d^	Adjusted mean difference^e^ (95% CI)
**Neurocognitive impairment (GDS)**^f^			p = 0.001	
No	843 (64.5)	65.17 (25.04)	ref	ref
Yes	463 (35.5)	60.55 (24.92)	-4.62 (-7.45 to -1.78)	-2.20 (-4.81 to 0.40)
**Gender at birth**			p < 0.001	
Male	1,008 (77.2)	65.00 (24.96)	ref	ref
Female	298 (22.8)	58.55 (24.92)	-6.45 (-9.67 to -3.22)	-6.53 (-9.57 to -3.49)
**Age (years)**			p < 0.001	
≤ 39	422 (32.3)	70.60 (23.83)	ref	ref
40–49	594 (45.5)	61.42 (24.65)	-9.19 (-12.25 to -6.12)	-6.32 (-4.28 to -9.21)
50–59	254 (19.4)	56.43 (24.96)	-14.17 (-18.00 to -10.35)	-10.49 (-14.-8 to -6.91)
≥ 60	36 (2.8)	65.50 (28.10)	-5.11 (-13.47 to 3.26)	-2.36 (-10.10 to 5.38)
**Race/Ethnicity**			p = 0.606	
Black/African American	605 (46.3)	62.79 (23.98)	ref	ref
White	544 (41.7)	63.69 (26.23)	0.90 (-2.01 to 3.81)	-4.45 (-7.22 to -1.68)
Hispanic	124 (9.5)	65.69 (24.30)	2.95 (-1.96 to 7.75)	0.51 (-3.93 to 4.96)
Other	33 (2.5)	66.22 (28.76)	3.42 (-5.38 to 12.23)	-3.21 (-11.21 to 4.79)
**Employment**			p < 0.001	
Full time	222 (17.0)	82.31 (17.45)	ref	ref
Part-time	163 (12.5)	68.66 (21.23)	-13.65 (-18.38 to -8.92)	-11.95 (-16.58 to -7.33)
Unemployed	921 (70.5)	58.09 (24.91)	-24.22 (-27.65 to -20.79)	-21.97 (-25.44 to -18.50)
**Lowest CD4 count (cells/mm**^**3**^**)**			p < 0.001	
≥ 500	121 (9.3)	72.77 (24.49)	ref	ref
200–499	481 (36.8)	65.74 (25.84)	-12.34 (-17.13 to -7.55)	-6.08 (-10.60 to -1.56)
< 200	704 (53.9)	60.43 (24.13)	-7.04 (-12.00 to -2.09)	-7.20 (-11.64 to -2.77)
**Current opiate test results**			p < 0.001	
Positive	95 (7.3)	48.61 (24.92)	ref	ref
Negative	1,211 (92.7)	64.70 (24.73)	16.09 (10.91 to 21.26)	11.12 (6.35 to 15.90)

^a^n, sample size with percentage of respondents in bracket; ref means the reference category;

^b^HRQoL, Health-related quality of life; Scores vary from 0–100 with higher scores indicating better quality of life;

^c^SD, Standard deviation;

^d^CI, Confidence interval;

^e^Adjusted for other covariates in the table using general linear model analysis;

^f^GDS, global deficit scores, used to determine impairment.

### Relationship between NCI and mental and physical HRQoL in adjusted analyses

Impaired participants had lower mental HRQoL compared to unimpaired participants in unadjusted analysis. The mean difference was lower when adjusted for age, gender at birth, race/ethnicity, unemployment, and current psychotropic medication use. In adjusted analysis, female gender, white and Hispanic race/ethnicity, current unemployment or part-time employment and current psychotropic medication use were associated with lower mental HRQoL (Table 2).

The negative association between NCI and physical HRQoL was detectable only in crude analysis and not when adjusted for gender, age, race/ethnicity, employment, lowest CD4 count and positive opiate test results. In adjusted analysis, female gender, white race/ethnicity, age group 40–59, unemployment, lowest CD4 below 500 cells/mm^3^ and positive opiate test results were associated with lower physical HRQoL (Table 3).

The Durbin-Watson test coefficients for the mental and physical HRQoL models were less than 2.0 indicating that the residuals were uncorrelated. Model fit, homogeneity of variance, normality and collinearity (variance inflation factor<1.2) were adequate.

In a separate linear regression model that included depression, the association between NCI and mental HRQoL reduced from -2.56 to -1.43 (95% CI = -2.89 to 0.03) and the coefficient of determination (R^2^) increased from 10.6% to 63.4%. Similarly, the association between NCI and physical HRQoL reduced from -2.20 to -0.60 (95% CI = -2.73 to 1.53) and the coefficient of determination (R^2^) increased from 20.0% to 47.0%.

### Mediating effect of depression

The crude ordinary least squares regression showed that NCI was negatively associated with depression (p = 0.020) and depression was negatively associated with mental (p < 0.001) and physical HRQoL (p < 0.001). Controlling for depression, NCI was associated with mental (p = 0.005) but not physical HRQoL (p = 0.07). The Sobel test indicated a discernible indirect effect of NCI on mental health (p = 0.020) and physical health (p = 0.009). The proportion of the total effect mediated was similar for physical health (51.2%) and for mental health (51.5%). These results suggest that depression partially mediated the relationship between HIV-associated NCI and mental and physical HRQoL

## Discussion

We systematically investigated the association between HIV-associated NCI, depression and HRQoL in a large and diverse sample of male and female PLWH in the US in the era of efficacious antiretroviral treatment. We used global deficit scores (GDS) to measure NCI in a large sample of male and female HIV-positive CHARTER participants. NCI was measured objectively via a comprehensive NP assessment of seven cognitive domains. To improve the validity of the measures, we excluded participants with comorbidities, including major depressive symptoms that confounded performance on the NP tests.

We found that NCI was associated with lower mental HRQoL. Our findings are generally consistent with previous reports of a negative association between NCI and mental HRQoL [8–12]. For example, a recent large study used structural equation modelling and found a direct association between depression and quality of life but an indirect relationship between cognitive performance and quality of life [11]. This study differs from ours because it was conducted in Canada using a male-only sample and utilized different measures of cognitive ability (the Brief Cognitive Ability Measure). Additionally, quality of life was measured by a single question. Parsons et al. found a relationship between poorer quality of life and lower neurocognitive function on selected tests; however, their results were based on a different quality of life instrument and a crude analysis of a small sample [13]. A US-based large study that utilized NP measures similar to those used in our study established a longitudinal relationship between transition to a more severe stage of cognitive impairment and declining HRQoL. The study also found that fewer symptoms of depression were associated with improved HRQoL. However, the study only included gay and bisexual men, and treated depression as a confounder of the relationship between NCI and HRQoL [12].

In contrast with previous studies that reported a negative association between NCI and physical HRQoL, we found that the presence of NCI was not associated with lower physical HRQoL [9] in adjusted analysis. The lack of detectable differences in physical HRQoL between impaired and unimpaired participants was likely because pain perception (p = 0.181) and general health perception (p = 0.160), which loaded on the physical health factor, did not differ between the two groups.

The study of Shrestha et al. conducted a moderated mediation analysis of the relationship between NCI and HRQoL [10]. Although, their study was limited to incarcerated HIV+ Malaysian males meeting the criteria for opioid dependence, it reported that depression may partially explain the association between NCI and HRQoL [10]. Thus, we conducted a mediation analysis. We found that depression indeed mediated the association between NCI and mental HRQoL, accounting for 51.5% of the effect, consistent with findings of previous studies [10, 24].

The 35-item MOS-HIV questionnaire has been used extensively in HIV/AIDS studies and has been shown to be a reliable and valid measure of HRQoL [14–16]. However, these studies have found inconsistent results relating to which scales can be grouped together to produce summary scores. A two-factor solution was confirmed by Revicki et al., but Taylor et al., reported a single factor structure for the MOS-HIV instrument when administered in Shona-speaking PLWH persons in Zimbabwe [14–16]. In the study by Taylor et al., one of the ten scales, “energy or vitality’, loaded on two factors [16]. In a multinational study to establish the reliability and validity of the MOS-HIV, Scott-Lennox et al. extracted two-factor solutions (physical and mental) for French, Italian, Dutch and UK translations of the MOS-HIV but a single factor solution for the German translation [15]. Thus, we factor analyzed the 35-item MOS-HIV questionnaire to verify that the questionnaire could be summarized into two dimensions, physical and mental HRQoL. We found that a one factor solution was admissible but a two factor solution was ideal for summarizing the MOS-HIV health survey questionnaire.

### Limitations

The study had some limitations. The CHARTER study was based on volunteers attending clinics at participating centers. This implies that the sample may not be representative of HIV-infected patients from other clinics or patients who did not volunteer for such studies. To maximize generalizability, the CHARTER study used minimal exclusion criteria to ensure the sample was as inclusive as possible of the HIV population visiting the study clinics. Data were collected using different methods involving patient self-reports for the outcome variable and a mixture of clinician ratings and self-report for the exposure variable. Thus, the data may be subject to mode effects. HIV-associated NCI was measured using GDS, which may not capture subtle forms of NCI [19]. Lastly, while the CHARTER study is one of the most comprehensive and analyzed datasets on NCI in HIV in the era of treatment, the data were collected from 2003 to 2007 and the current HIV population may be different.

## Conclusion

We had hypothesized that NCI is associated with lower physical and mental HRQoL. We found lower (worse) mental HRQoL scores among neurocognitively impaired HIV-infected participants. However, the lower bound on the error of estimation for the mean adjusted difference in mental HRQoL between impaired and unimpaired participants was negative but close to zero indicating that this finding may not be clinically important. Although the adjusted difference is unlikely to be due to chance alone, how big the difference is clinically cannot be ascertained from this result. Further studies are suggested to determine a minimum detectable difference in health related quality of life between impaired and unimpaired PLWH that can be regarded as medically significant.

Depression partially mediated the association between NCI and HRQoL. These findings suggest that future strategies aimed at improving HRQoL among PLWH with NCI might benefit from concurrent management of depression.

We found that a two-factor solution was ideal for summarizing the MOS-HIV health survey questionnaire into mental and physical HRQoL. This finding may provide guidance on how to combine the different scales of the MOS-HIV questionnaire in future studies.

## Supporting information

S1 Fig Directed acyclic graph (DAG) showing the hypothesized relationship among study variables. Reference for DAG, http://www.dagitty.net/experimental/dags.html. HAART, Highly active antiretroviral therapy; NCI, HIV-associated neurocognitive impairment; HRQoL, Health related quality of life; CNS Integrity, Central nervous system integrity represented by HIV viral load in plasma, HIV viral load in cerebrospinal fluid (CSF), CSF total protein and CSF glucose.
(TIF)

S1 Table CHARTER^a^ study neuropsychological test battery by cognitive domain. ^a^CHARTER, Central nervous system HIV antiretroviral therapy effects research.
(DOCX)

S2 Table Characteristics of the CHARTER study participants and mean scores on mental and physical health components of the Medical Outcome Survey Questionnaire (n = 1587). ^a^n, Sample size; ^b^SD, Standard deviation; ^c^ANI, asymptomatic neurocognitive impairment; ^c^HAD, HIV associated neurocognitive disorder; ^c^MND, Mild neurocognitive impairment; NP-NML, neuropsychologically normal; ^d^ARV, antiretroviral; ^e^HAART, Highly active antiretroviral therapy.
(DOCX)

S3 Table Differences in mental health-related quality of life (HRQoL) between IV neurocognitive impaired and unimpaired CHARTER^a^ study subjects. ^a^CHARTER, Central nervous system (CNS) HIV antiretroviral therapy effects research study; ^b^SD, Standard deviation; ^c^p-value based on t-test; ^d^Mental HRQoL composite scores were computed as factor-based scores by adding the average HRQoL scores on all preceding scales that loaded on same factor.
(DOCX)

S4 Table Differences in physical health-related quality of life (HRQoL) between HIV neurocognitive impaired and unimpaired CHARTER^a^ study subjects. ^a^CHARTER, Central nervous system (CNS) HIV antiretroviral therapy effects research study; ^b^SD, Standard deviation; ^c^p-value based on t-test; ^d^Physical HRQoL composite scores were computed as factor-based scores by adding the average HRQoL scores on all preceding scales that loaded on same factor.
(DOCX)
