# Supplementary figures and images for: Neurocognitive impairment and health-related quality of life among people living with Human Immunodeficiency Virus (HIV)
Source: PLoS One. 2021 Apr 1;16(4):e0248802. doi: 10.1371/journal.pone.0248802 (PMC8016250; doi:10.1371/journal.pone.0248802)

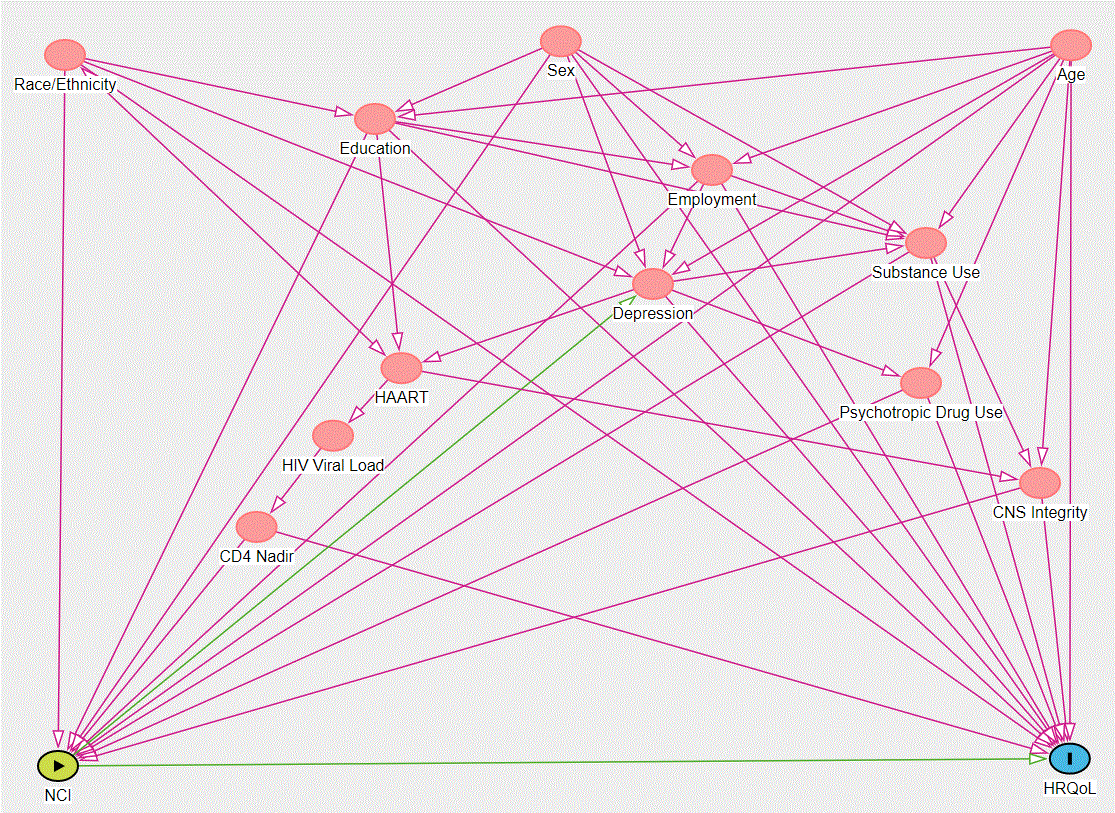

Supplement: S1 Fig — Reference for DAG, http://www.dagitty.net/experimental/dags.html. HAART, Highly active antiretroviral therapy; NCI, HIV-associated neurocognitive impairment; HRQoL, Health related quality of life; CNS Integrity, Central nervous system integrity represented by HIV viral load in plasma, HIV viral load in cerebrospinal fluid (CSF), CSF total protein and CSF glucose. (TIF) [file pone.0248802.s001.tif]
