# Supplementary material for: Neurocognitive impairment and health-related quality of life among people living with Human Immunodeficiency Virus (HIV)
Source: PLoS One. 2021 Apr 1;16(4):e0248802. doi: 10.1371/journal.pone.0248802 (PMC8016250; doi:10.1371/journal.pone.0248802)
Supplement: S1 Table — aCHARTER, Central nervous system HIV antiretroviral therapy effects research. (DOCX) [file pone.0248802.s002.docx]

**S1 Table. CHARTER^a^ study neuropsychological test battery by cognitive domain.**

| **Domain** | **Test** |
| --- | --- |
| **1. Speed of information processing** | Wechsler adult intelligence scale [WAIS]-II -digit symbol |
|  | Wechsler adult intelligence scale (WAIS-III) -symbol search |
|  | Trail making test part A |
| **2. Learning** | Hopkins verbal learning tests revised [HVLT-R]-learning trials |
|  | Brief visuospatial memory test revised [BVMT-R]-learning trials |
|  | Story memory test (learning component) |
|  | Figure memory test (learning component) |
| **3. Memory** | Hopkins verbal learning test-revised (HVLT-R)-delayed recall |
|  | Brief visuospatial memory test-revised (BVMT-R)-delayed recall |
|  | Story memory test (with delayed recall) |
|  | Figure Memory Test (delayed recall component); |
|  | Brief visuospatial memory test revised, |
| **4. Executive function** | Category fluency test |
|  | Wisconsin card sorting computerized test [64-item version], |
|  | Trail making test part B |
| **5. Verbal fluency** | Verbal fluency-controlled oral word association test [F-A-S letters] |
|  | Category fluency test [animals] |
| **6. Attention and working**  **memory** | Paced auditory serial addition test-50 |
|  | WAIS-III letter-number sequencing |
| **7. Motor function** | Grooved pegboard test, dominant and non-dominant hands |

^a^CHARTER, Central nervous system HIV antiretroviral therapy effects research.
