# Supplementary material for: Neurocognitive impairment and health-related quality of life among people living with Human Immunodeficiency Virus (HIV)
Source: PLoS One. 2021 Apr 1;16(4):e0248802. doi: 10.1371/journal.pone.0248802 (PMC8016250; doi:10.1371/journal.pone.0248802)
Supplement: S2 Table — an, Sample size; bSD, Standard deviation; cANI, asymptomatic neurocognitive impairment; cHAD, HIV associated neurocognitive disorder; cMND, Mild neurocognitive impairment; NP-NML, neuropsychologically normal; dARV, antiretroviral; eHAART, Highly active antiretroviral therapy. (DOCX) [file pone.0248802.s003.docx]

| **S2 Table. Characteristics of the CHARTER study participants and mean scores on mental and physical health components of the Medical Outcome Survey (MOS) questionnaire (n=1587).** | | | | |
| --- | --- | --- | --- | --- |
| **Characteristics** | **n^a^** | **Percent** | **Mental health-related quality of life scores** | **Physical health-related quality of life scores** |
|  |  |  | **Mean (SD)^b^** | **Mean (SD)^b^** |
| **Gender at Birth** |  |  |  |  |
| Male | 1216 | 76.62 | 66.52 (18.37) | 63.8 (24.87) |
| Female | 371 | 23.38 | 63.00 (20.01) | 57.57 (24.42) |
| **Ethnicity** |  |  |  |  |
| Black or African American | 766 | 48.27 | 67.73 (18.00) | 61.98 (23.68) |
| White | 631 | 39.76 | 63.74 (19.44) | 62.54 (26.18) |
| Hispanic | 149 | 9.39 | 63.15 (18.51) | 62.73 (25.01) |
| Other | 41 | 2.58 | 67.31 (21.37) | 64.72 (27.16) |
| **Age (years)** |  |  |  |  |
| <=39 | 496 | 31.25 | 65.92 (19.34) | 68.46 (24.56) |
| 40-49 | 745 | 46.94 | 65.58 (18.49) | 61.04 (24.34) |
| 50-59 | 306 | 19.28 | 64.86 (18.53) | 55.49 (24.67) |
| >=60 | 40 | 2.52 | 71.69 (20.03) | 63.23 (27.99) |
| **Education** |  |  |  |  |
| Higher school diploma or less | 704 | 44.73 | 64.22 (18.71) | 60.68 (24.53) |
| Associate degree, or some college | 603 | 38.31 | 66.61 (18.84) | 61.75 (25.01) |
| Undergraduate Degree or higher | 267 | 16.96 | 67.8 (18.83) | 68.06 (25.22) |
| **Employment** |  |  |  |  |
| Full time | 236 | 14.87 | 74.63 (16.23) | 82.63 (17.18) |
| Part-time | 191 | 12.04 | 66.56 (18.17) | 68.16 (21.12) |
| Not currently employed | 1160 | 73.09 | 63.74 (18.89) | 57.26 (24.50) |
| **Neurocognitive impairment** |  |  |  |  |
| Impaired | 659 | 41.58 | 63.53 (18.36) | 59.69 (24.21) |
| Not impaired | 926 | 58.42 | 67.34 (18.89) | 64.31 (25.19) |
| **HAND diagnosis^c^** |  |  |  |  |
| ANI | 453 | 32.75 | 69.38 (16.80) | 66.63 (23.15) |
| HAD | 33 | 2.39 | 53.48 (12.94) | 41.38 (22.66) |
| MND | 140 | 10.12 | 50.72 (16.90) | 47.19 (22.99) |
| NP-NML | 757 | 54.74 | 67.91 (18.71) | 64.96 (25.08) |
| **Highly active antiretroviral therapy^d^** |  |  |  |  |
| ARV naive | 255 | 16.14 | 67.94 (18.60) | 71.28 (24.22) |
| HAART | 1003 | 63.48 | 65.37 (19.09) | 61.02 (24.48) |
| No current ARVs | 214 | 13.54 | 63.67 (18.14) | 57.85 (25.24) |
| Non-HAART | 108 | 6.84 | 68.14 (17.91) | 62.85 (25.58) |
| **ARV history** |  |  |  |  |
| ARV Naive | 255 | 16.14 | 67.94 (18.60) | 71.28 (24.22) |
| Current ARV use | 1111 | 70.32 | 65.64 (18.99) | 61.19 (24.58) |
| Past ARV use | 214 | 13.54 | 63.67 (18.14) | 57.85 (25.24) |
| **Value of lowest CD4 count (cells/mm per cubic)** | |  |  |  |
| <200 | 870 | 55.38 | 65.35 (18.85) | 59.72 (24.02) |
| 200-499 | 563 | 35.84 | 65.74 (18.70) | 64.42 (25.62) |
| >=500 | 138 | 8.78 | 68.76 (18.83) | 70.28 (25.69) |
| **Depressive symptoms** |  |  |  |  |
| Minimal | 880 | 55.87 | 77.24 (12.09) | 73.88 (20.69) |
| Mild | 249 | 15.81 | 59.79 (13.29) | 54.87 (22.32) |
| Moderate | 264 | 16.76 | 50.82 (12.41) | 47.32 (21.03) |
| Severe | 182 | 11.56 | 40.33 (14.93) | 38.93 (19.47) |
| **Lifetime alcohol use or dependence** |  |  |  |  |
| No | 703 | 44.78 | 66.88 (19.03) | 63.86 (25.15) |
| Yes | 867 | 55.22 | 65.00 (18.57) | 61.34 (24.65) |
| **Opiate test results** |  |  |  |  |
| Negative | 1429 | 91.78 | 66.23 (18.77) | 63.85 (24.68) |
| Positive | 128 | 8.22 | 59.49 (19.28) | 47.37 (23.99) |
| **Hepatitis C antibody lab results** |  |  |  |  |
| Negative | 1144 | 73.95 | 66.02 (18.64) | 64.07 (24.78) |
| Positive | 403 | 26.05 | 65.59 (19.51) | 58.79 (25.18) |
| **Clinical neuropathy severity grading** |  |  |  |  |
| No neuropathy | 270 | 31.69 | 65.82 (19.21) | 63.02 (25.03) |
| Mild | 356 | 41.78 | 65.65 (18.18) | 57.29 (24.55) |
| Moderate | 161 | 18.9 | 64.61 (20.20) | 56.02 (24.19) |
| Severe | 65 | 7.63 | 58.25 (17.42) | 44.81 (22.74) |
| **Comorbidity rating** |  |  |  |  |
| Confounding | 247 | 15.56 | 61.31 (19.48) | 56.70 (23.84) |
| Contributing | 476 | 29.99 | 62.36 (18.49) | 56.61 (24.42) |
| Incidental | 864 | 54.44 | 68.80 (18.25) | 67.12 (24.48) |

^a^n, Sample size; ^b^SD, Standard deviation; ^c^ANI, asymptomatic neurocognitive impairment; ^c^HAD, HIV associated neurocognitive disorder; ^c^MND, Mild neurocognitive impairment; NP-NML, neuropsychologically normal; ^d^ARV, antiretroviral; ^e^HAART, Highly active antiretroviral therapy.
