# Supplementary material for: Neurocognitive impairment and health-related quality of life among people living with Human Immunodeficiency Virus (HIV)
Source: PLoS One. 2021 Apr 1;16(4):e0248802. doi: 10.1371/journal.pone.0248802 (PMC8016250; doi:10.1371/journal.pone.0248802)
Supplement: S3 Table — aCHARTER, Central nervous system (CNS) HIV antiretroviral therapy effects research study; bSD, Standard deviation; cp-value based on t-test; dMental HRQoL composite scores were computed as factor-based scores by adding the average HRQoL scores on all preceding scales that loaded on same factor. (DOCX) [file pone.0248802.s004.docx]

| **S3 Table. Differences in mental health-related quality of life (HRQoL) between HIV neurocognitive impaired and unimpaired CHARTER^a^ study subjects.** | | | | | |
| --- | --- | --- | --- | --- | --- |
| **Scales** | **Impaired**  **(n = 386)** | **Unimpaired**  **(n = 679)** | p-value^c^ | Pooled SD | Cohen's D^d^ |
|  | Mean (SD)^b^ | Mean (SD) |  |  |  |
| Mental health | 62.35 (21.36) | 66.21 (21.72) | 0.005 | 21.59 | 0.18 |
| Energy/Fatigue | 48.45 (22.26) | 52.63 (22.18) | 0.003 | 22.21 | 0.19 |
| Health distress | 68.13 (25.62) | 73.02 (25.97) | 0.003 | 25.85 | 0.19 |
| Cognitive function | 67.03 (24.30) | 73.99 (22.70) | <0.001 | 23.29 | 0.30 |
| Quality of life | 61.27 (22.56) | 63.25 (21.62) | 0.156 | 21.96 | 0.09 |
|  |  |  |  |  |  |
| Mental HRQoL composite^e^ | 61.45 (18.12) | 65.82 (18.82) | <0.002 | 18.56 | 0.24 |

^a^CHARTER, Central nervous system (CNS) HIV antiretroviral therapy effects research study; ^b^SD, Standard deviation; ^c^p-value based on t-test; ^d^Cohen's D was determined by calculating the mean difference between unimpaired and impaired respondents, and then dividing the result by the pooled standard deviation (SD) computed from a two-sample independent t-test; ^e^Mental HRQoL composite scores were computed as factor-based scores by adding the average HRQoL scores on all preceding scales that loaded on same factor
