# Supplementary material for: Neurocognitive impairment and health-related quality of life among people living with Human Immunodeficiency Virus (HIV)
Source: PLoS One. 2021 Apr 1;16(4):e0248802. doi: 10.1371/journal.pone.0248802 (PMC8016250; doi:10.1371/journal.pone.0248802)
Supplement: S4 Table — aCHARTER, Central nervous system (CNS) HIV antiretroviral therapy effects research study; bSD, Standard deviation; cp-value based on t-test; dPhysical HRQoL composite scores were computed as factor-based scores by adding the average HRQoL scores on all preceding scales that loaded on same factor. (DOCX) [file pone.0248802.s005.docx]

| **S4 Table. Differences in physical health-related quality of life (HRQoL) between HIV neurocognitive impaired and unimpaired CHARTER^a^ study subjects.** | | | | | |
| --- | --- | --- | --- | --- | --- |
| **Scales** | **Impaired**  **(n = 463)** | **Unimpaired**  **(n = 843)** | **p-value^c^** | Pooled SD | Cohen’s D^d^ |
|  | Mean (SD)^b^ | Mean (SD) |  |  |  |
| Pain | 65.59 (27.75) | 67.66 (26.15) | 0.181 | 26.7 | 0.08 |
| Physical function | 65.85 (27.94) | 72.70 (26.82) | <0.001 | 27.2 | 0.25 |
| Role function | 50.97 (45.63) | 58.60 (44.99) | 0.004 | 45.2 | 0.17 |
| Social function | 72.35 (28.63) | 76.68 (27.39) | 0.007 | 27.8 | 0.16 |
| General health | 47.99 (25.86) | 50.19 (27.77) | 0.16 | 27.1 | 0.08 |
|  |  |  |  |  |  |
| Physical HRQoL composite^e^ | 60.55 (24.92) | 65.17 (25.04) | 0.001 | 25.0 | 0.18 |

^a^CHARTER, Central nervous system (CNS) HIV antiretroviral therapy effects research study; ^b^SD, Standard deviation; ^c^p-value based on t-test; ^d^Cohen's d was determined by calculating the mean difference between unimpaired and impaired respondents, and then dividing the result by the pooled standard deviation (SD) computed from a two-sample independent t-test; ^e^Physical HRQoL composite scores were computed as factor-based scores by adding the average HRQoL scores on all preceding scales that loaded on same factor.
